# Design and rationale of a matched cohort study to assess the effectiveness of a combined household-level piped water and sanitation intervention in rural Odisha, India

**DOI:** 10.1136/bmjopen-2016-012719

**Published:** 2017-03-31

**Authors:** Heather Reese, Parimita Routray, Belen Torondel, Gloria Sclar, Maryann G Delea, Sheela S Sinharoy, Laura Zambrano, Bethany Caruso, Samir R Mishra, Howard H Chang, Thomas Clasen

**Affiliations:** 1 Department of Environmental Health, Rollins School of Public Health, Emory University, Atlanta, Georgia, USA; 2 London School of Hygiene and Tropical Medicine, London, UK; 3 Nutrition and Health Sciences Program, Laney Graduate School, Emory University, Atlanta, Georgia, USA; 4 School of Biotechnology, KIIT University, Bhubaneswar, Odisha, India; 5 Department of Biostatistics and Bioinformatics, Rollins School of Public Health, Emory University, Atlanta, Georgia, USA

**Keywords:** sanitation, piped water supply, diarrheal diseases

## Abstract

**Introduction:**

Government efforts to address massive shortfalls in rural water and sanitation in India have centred on construction of community water sources and toilets for selected households. However, deficiencies with water quality and quantity at the household level and community coverage and actual use of toilets have led Gram Vikas, a local non-governmental organization in Odisha, India, to develop an approach that provides household-level piped water connections contingent on full community-level toilet coverage.

**Methods:**

This matched cohort study was designed to assess the effectiveness of a combined piped water and sanitation intervention. Households with children <5 years in 45 randomly selected intervention villages and 45 matched control villages will be followed over 17 months. The primary outcome is prevalence of diarrhoeal diseases; secondary health outcomes include soil-transmitted helminth infection, nutritional status, seroconversion to enteric pathogens, urogenital infections and environmental enteric dysfunction. In addition, intervention effects on sanitation and water coverage, access and use, environmental fecal contamination, women's empowerment, as well as collective efficacy, and intervention cost and cost-effectiveness will be assessed.

**Ethics and dissemination:**

The study protocol has been reviewed and approved by the ethics boards of the London School of Hygiene and Tropical Medicine, UK and KIIT University, Bhubaneswar, India. Findings will be disseminated via peer-reviewed literature and presentation to stakeholders, government officials, implementers and researchers.

**Trial registration number:**

NCT02441699.

**Strengths and limitations of this study**

- The study assesses a combined household-level piped water and sanitation intervention that requires complete community-level compliance.
- The intervention was not randomly allocated; but, controls are selected through a restriction process to limit possible partial exposure to the intervention through spill over, and matched to intervention villages using preintervention data.
- The study uses a holistic definition of health to assess intervention impacts on physical, mental and social well-being, including more novel outcomes such as seroconversion to enteric pathogens, environmental enteric dysfunction and sanitation insecurity. It also assesses intervention coverage, cost-effectiveness and collective efficacy.
- The time lapse between intervention completion and the beginning of the evaluation process prevents baseline comparison or assessment of immediate intervention impacts. However, it allows for a biologically plausible length of time for die-off of even the most persistent pathogens in the environment and provides time for children to have been born into this environment.

## Introduction

Of the one billion people practicing open defecation worldwide, over half of them live in India.^1^ While international and national pressure on improving sanitation conditions in India has led to over 350 000 people gaining access to improved toilets since 1990, it has barely kept up with population growth.^1^ ^2^ Recent studies show that even in areas with access to household-level improved sanitation, use of these toilets is low.^3–5^ This may be due in part to a mismatch between the culturally acceptable pour-flush toilets and the level of water access. Coverage of improved water sources, usually community-level pumps or taps, is relatively high even in rural areas in India, but it may not be sufficient for flushing purposes on top of other daily water needs.^1^ ^6^

Although the effectiveness of water, sanitation and hygiene (WASH) interventions vary, meta-analyses have found that individual or combined WASH interventions decrease diarrhoeal disease prevalence by up to 48%.^7–11^ While combined interventions would be expected to have a greater influence on multiple exposure pathways and thus a greater combined impact on health, there is limited evidence of additive benefits.^12^ This may be due to poor uptake, inconsistent use or an incomplete understanding of relevant pathways.^8–10^ In India, combining water and sanitation interventions may be more critical than just interrupting multiple transmission pathways for enteric infection; evidence suggests that household-level water access is integral to the use of improved sanitation in this context.^13^

While the intent of improved sanitation facilities is to separate human feces from human contact, most of the focus is on constructing household toilets to increase improved sanitation coverage—the primary metric used in monitoring progress towards international targets. However, studies in India have further shown that toilet construction does not translate into toilet use in this context.^5^ ^14–16^ Moreover, with the interdependence between members of households and households within communities, safe water and sanitation is a community-level issue. There is growing emphasis on assessing health risk from poor water and sanitation conditions not simply due to individual or even household-level risk factors, but also from conditions in the community environment.^17^ There is evidence that even households without toilets, and households which do not filter drinking water, showed decreased health risk if they live in communities with high levels of coverage and use.^18–20^

Moreover, the effectiveness of community interventions may be higher in communities with positive perceptions of their collective ability to come together to improve their conditions. Collective efficacy (CE), a latent construct comprised the structural and cognitive components that facilitate a community's shared belief in its ability to come together and execute actions related to a common goal, may explain some variance in intervention effectiveness across communities receiving WASH interventions.^21^

A main risk of poor WASH conditions is enteric infection, caused by a diverse array of bacteria, viruses, protozoa and parasites, including soil-transmitted helminths. These infections may cause diarrhoea, the second leading cause of mortality for children <5 years worldwide and in India, a leading cause of mortality regardless of age.^22^ ^23^ There is also growing evidence that asymptomatic enteric infections may pose a similar risk, with repeat enteric infections contributing to chronic malnutrition, environmental enteric dysfunction (EED), poor cognitive outcomes and poor vaccine uptake.^24–29^ Poor WASH conditions are also linked to increased risk of respiratory infection, the leading cause of mortality for children <5 years worldwide.^22^ ^30^ ^31^ Poor water and sanitation access can also affect the social, physical and mental well-being of women, acting through pathways ranging from unsafe menstrual hygiene management practices and increased risk of violence.^32–34^

### Description of the intervention

Over the past few decades, there has been a global commitment to determine water and sanitation interventions with demonstrated effectiveness, not just efficacy.^35^ Gram Vikas, a non-governmental organisation based in Odisha, India (http://www.gramvikas.org/), has responded by implementing its MANTRA (Movement and Action Network for Transformation of Rural Areas) water and sanitation programme in more than 1000 villages since 2002.^36^ This approach includes household-level piped water connections and community-level mobilisation for culturally appropriate household toilets. A previous interrupted time series analysis of the MANTRA intervention reported it to be protective against diarrhoeal diseases.^37^ However, in addition to limitations of design, this study relied on outcome data collected and reported by Gram Vikas, the intervention implementer, and did not assess intervention coverage or impacts on environmental fecal contamination.

The MANTRA water and sanitation intervention is rolled out in a three-phase process over an average of 3 years. During the first, or Motivational, phase (∼8–12 months), representatives of Gram Vikas visit the identified village several times to assess village interest and progress towards a set of Gram Vikas requirements, including: (1) the commitment of every household to participate, (2) creation of a village corpus fund from contributions from every household and (3) development of village guidelines for maintenance and use of facilities.

Once this set of requirements is achieved, the village progresses into the second, or Operational, phase of the intervention (∼17–35 months). Each household constructs a pour-flush toilet with two soak-pits and a separate bathing room. The households hire a local, skilled mason and provide their own unskilled labour and locally available materials to complete the superstructure. Gram Vikas provides external materials such as PVC pipes and porcelain pans. At the same time, a water tank, community meeting space and piped water distribution system connected to every household, with taps in the toilet and bathing rooms and a separate tap in the kitchen, is constructed through a similar collaborative process.

All households must construct a toilet and bathing room for the village to progress into the final, or completed, phase of the intervention, in which the water system is turned on. Notably, this three-phase process only allows each household access to piped water once every household in the village has a toilet and bathing room. This model contrasts with most previous water and sanitation interventions, including those implemented under India's Total Sanitation Campaign and other government programmes, which do not require community-level sanitation compliance and do not provide a piped water supply at the household level.^38^

### Study aims

The primary objective of this study is to evaluate the effectiveness of the combined household-level water supply and sanitation intervention, as implemented by Gram Vikas in Odisha, India. Towards that objective, this study aims to:

1. Assess the effectiveness of the intervention in improving water and sanitation infrastructure coverage, access, and use, and to assess fecal sludge management practices in intervention communities.
2. Assess the effectiveness of the intervention in reducing environmental fecal contamination.
3. Assess the effectiveness of the intervention in improving health. This includes reported diarrhoeal disease in children <5 years (primary outcome), acute respiratory infection, infection with soil-transmitted helminthes, nutritional status, EED, seroconversion for selected enteric pathogens and urogenital diseases associated with menstrual hygiene management practices. Mental and social well-being will be explored through assessment of sanitation insecurity and women's empowerment.
4. Assess the cost and cost-effectiveness of the intervention.
5. Develop and assess a theoretically grounded, empirically informed CE scale and determine the effect of CE on intervention effectiveness.

## Methods

### Setting

**Figure 1:**
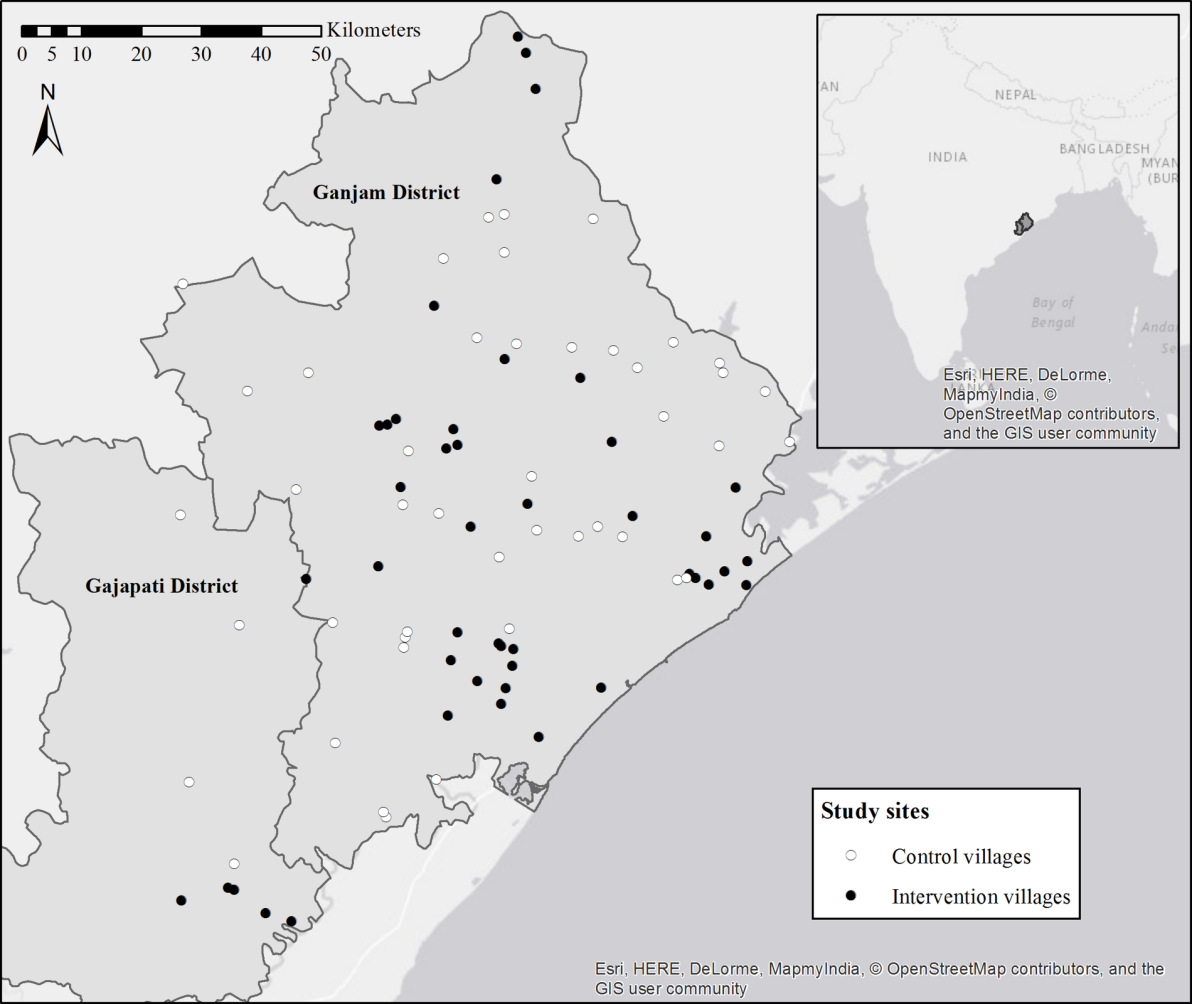
Study sites in Ganjam and Gajapati districts, Odisha, India, with intervention villages in black and control villages in white. Inset shows location of districts in India.

The study is located in Ganjam and Gajapati districts in eastern Odisha, India (figure 1). These two contiguous districts were a single district until 1992. Over 44% of the population in these districts is recognised by the Government of India as being below the poverty line (BPL).^39^ As of 2008, a majority of households in both districts had access to an improved, likely community-level, drinking water source, with over 23% of households in Ganjam having access to any sanitation facility, compared to only 8% of households in Gajapati.^39^ The area is primarily rural and agrarian, and the climate is characterised by a monsoon season from June to September, with an average rainfall of ∼1400 mm/year.

### Study design

**Figure 2:**
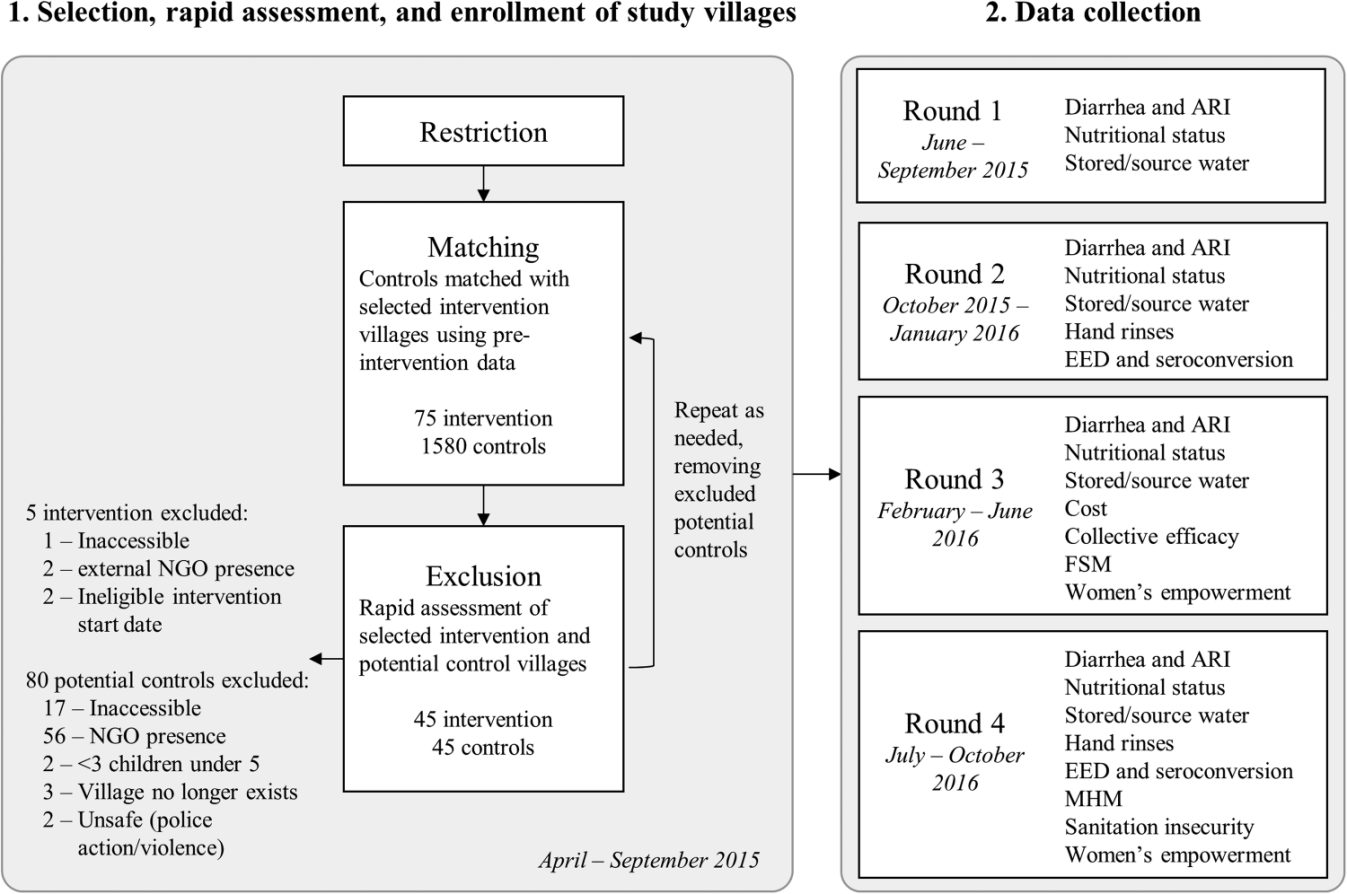
Restriction, matching and exclusion process for selection of intervention and control villages (1), and timeline for study rounds and outcome data collection (2).

This study uses a matched cohort design to assess the effectiveness of a completed intervention with data collected across four study rounds from June 2015 to October 2016 (figure 2). Data were collected in all study rounds for diarrhoea, acute respiratory infection, nutritional status and stored and source water outcomes to assess seasonality. Data were collected in rounds 2 and 4 for EED, seroconversion, and hand-rinses, and cross-sectionally in one or more rounds for the remaining outcomes. As described below, control villages were matched to randomly selected intervention villages through a multistep restriction, genetic matching and exclusion process using the following eligibility criteria.

### Eligibility criteria for villages

#### Restriction

Intervention villages were randomly selected from a list of Gram Vikas villages in Ganjam and Gajapati districts provided by the non-governmental organization (NGO), after restriction to villages with a Motivation phase start date between 2002 and 2006 and a Construction phase start date no earlier than 2003. Since the intervention process takes on average 3 years, the criteria for the Motivation start date helped to identify those villages with ongoing interventions at the same time. In addition, this allowed the use of the Government of India Census 2001 and the BPL Survey 2002 data to characterise baseline characteristics used in the matching process in intervention and control villages.

Eligible control villages include all villages without a Gram Vikas intervention within the study districts which: (1) are not within the same Gram Panchayat (a political subdivision with some administrative responsibility for water and sanitation comprised several villages) as a Gram Vikas village, or bordering a Gram Vikas village, and (2) had not received a Motivation visit from the Gram Vikas NGO. These criteria serve to limit the possibility of previous partial exposure to the intervention through spill over from adjacent villages or direct contact with the NGO. These criteria also increase the strength of the comparison provided by the control villages, that is, it increases the likelihood that if they had received a motivation visit from Gram Vikas, the control villages would have been equally as likely as the intervention villages to demand the intervention.

In addition, to be eligible for inclusion intervention and control villages must (1) appear in the Government of India Census 2001 and the BPL Survey 2002, (2) have a population of at least 20 households and (3) be within ∼3 hours travel from the study office in Brahmapur, Ganjam District. This last criterion is due to logistical constraints.

#### Matching

After restriction, genetic matching was used to match potential control villages to the randomly selected intervention villages without replacement.^5^ ^40^ ^41^ Villages were exact matched on district to limit any political or large-scale geographic variation between district populations and were also matched on preintervention demographic, socioeconomic, sanitation and water access characteristics listed in table 1.^5^ These village-level matching variables were selected due to their theorised association with the primary outcome, diarrhoeal diseases, as well as data availability.

**Table 1:** Preintervention characteristics used in matching, and balance diagnostics before and after matching and exclusion process

	Intervention	Control (all eligible)	Std diff (all eligible)	Control (study)	Std diff (study)
Variable	(n=45)	(n=1580)		(n=45)	
Number of households	157.9	215.5	0.37	148.1	0.06
Population <6 years (%)	16.2	16.9	0.19	16.3	0.02
Household income score (x̅)	2.9	3.1**	0.26	2.9	0.01
Household goods owned (x̅)	1.1	1.2*	0.27	1.1	0.02
Pucca house (%)	59.2	61.6	0.09	60.5	0.05
≥2 meals a day (%)	57.7	63.7	0.19	57.8	0.01
Scheduled caste (%)	11.5	18.7**	0.46	11.8	0.01
Scheduled tribe (%)	33.4	19.1*	0.31	29.8	0.08
Female literacy (%)	30.9	29.8	0.07	30.9	0.00
Open defecation (%)	95.6	95.2*	0.04	95.8	0.01
Improved drinking water source† (%)	38.6	42.5	0.10	37.2	0.02
Water source <500 m and 50 m elevation (%)	81.5	72.2	0.31	81.7	0.01

All eligible: all villages that are eligible for the matching process after restriction.

Std diff (absolute standardised difference): a value >0.1 is considered meaningful imbalance.^42^

Kolmogorov-Smirnov bootstrap p values: *<0.05, **<0.01.

†Ganjam villages only; no data available for Gajapati villages.

#### Exclusion

The field team visited matched potential control villages and intervention villages to assess suitability for the study through a rapid assessment interview with village leadership and to ensure accessibility. Villages were excluded if they are not within 3 hours travel of the field office in Brahmapur, had sustained major infrastructure damage due to a natural disaster, or if there was a current or planned sanitation or water intervention by an organisation external to the village in the next 12 months as determined through the rapid assessment interview with village leadership. In addition, villages were excluded if there were fewer than three households with children <5 years old. As villages were removed from the pool of prospective control villages, the matching process was repeated for all intervention villages and remaining eligible control villages, and balance measures were assessed. The matching and exclusion processes were repeated as necessary.

After the iterative matching and exclusion process was complete, covariate balance was assessed for all matching variables for the final set of intervention and control villages through examination of balance measures.^42–44^ Matching resulted in an improvement in balance as assessed through comparison of several measures including q-q plots, Kolmogorov-Smirnov bootstrap p values and standardised differences. After matching, there were no significant differences between the intervention and control groups (table 1).

### Eligibility criteria for households

Households within selected intervention and control villages were eligible if they had at least one child <5 years at the time of enrolment, verified with birth or immunisation card and expected to reside in the village for the duration of the study. If there were more than 40 eligible households within a village, only 40 were randomly selected to be enrolled. Informed consent was obtained from the male and/or female head of the selected households. All children <5 years within each enrolled household were eligible and do not age-out over the course of the study. Households with newborn children were enrolled as they became eligible on an ongoing basis throughout the study, in villages with <40 enrolled households.

### Sample size

Sample size was determined through a simulation estimating the log odds of diarrhoeal disease (the primary outcome) through a multilevel random effects model and parameterised with data from a previous study in a neighbouring district in Odisha.^16^ Sample size estimates were also checked with G*Power.^45^ The simulation assumes a longitudinal 7-day period prevalence for diarrhoea of 8.8% in children <5 years, a heterogeneity variance between villages of 0.07, a heterogeneity variance between households of 0.57 and four study rounds.^16^ An effect size of 0.20 was selected for public health importance and based on estimates of effect from systematic reviews of water and sanitation studies.^46^ Assuming at least 80% power, 0.05 significance level, 10% for loss to follow-up and at least one child per household, we estimate a sample size of 45 villages per study arm and 26 households per village. This estimate was the most conservative compared with sample size estimates for secondary outcomes and was therefore used for the broader study population.

### Outcome measurement

Outcomes, and individual, household, and community-level risk factors, will be measured through surveys, interviews or through the collection and analysis of environmental, stool or dried blood spot samples. All survey questions will be translated into the primary local language, Odia, and back-translated to confirm wording. Household surveys include household and individual factors and will be verbally administered by trained field workers to the mother or primary caregiver of the youngest child <5 in each household, unless otherwise specified below. Community surveys will be verbally administered to the *sarpanch* (village head) or any other member of village leadership. Survey data will be collected on mobile phones using Open Data Kit.^47^ GPS coordinates for households, water sources and other relevant sites will be collected using Garmin eTrex 10 or 20 devices (Garmin, Olathe, Kansas, USA).

#### Coverage, access and use of sanitation, water and hygiene infrastructure

Coverage, access and use of WASH infrastructure will be assessed in all four rounds. Presence of and access to toilets, water sources and hand-washing stations will be assessed through standard questions from the Demographic and Health Surveys (DHS) and confirmed through spot observations. Spot observations of household toilets and hand-washing stations will be further used to assess indicators of functionality, maintenance, recent use. Reported water and sanitation practices, including child feces disposal practices, will be captured through household survey questions.

#### Diarrhoeal diseases

The primary outcome for this study is prevalence of diarrhoeal diseases, recorded as both daily point prevalence over the previous 3 days and 7-day period prevalence, for all household members in all four rounds. Although self-reported diarrhoea is a subjective outcome with a well-established risk of bias, three-day recall reduces recall bias.^48^ ^49^ Diarrhoeal disease will be measured using the WHO definition of three or more loose stools in a 24-hour period, with or without the presence of blood. Field workers will use a simple calendar as a visual aid to help respondents with recall. Each household member will be asked to recall his or her own disease status, and the mother or primary caregiver will be asked to report disease for children.

#### Respiratory infection

Prevalence of respiratory infections will be recorded as both daily point prevalence over the previous 3 days and 7-day period prevalence for all household members in all four rounds. Respiratory infection is defined as the presence of cough and/or shortness of breath/difficulty breathing according to WHO's Integrated Management of Childhood Illness (IMCI).^50^ The full IMCI case definition for acute lower respiratory infection also includes measurement of respiratory rate and observation of chest indrawing, stridor and other danger signs; these criteria were excluded from our definition as there was concern about the technical support required to produce consistent and accurate data within this context.^50^ Our definition provides a broad assessment of respiratory illness burden. Each household member will be asked to recall his or her own disease status, and the mother or primary caregiver will be asked to report disease for children.

#### Nutritional status

Anthropometric data will be collected for children under the age of five in all four rounds using standard methods as established by WHO.^51^ ^52^ Field workers will be trained and standardised in line with WHO protocols to reduce measurement error.^52^ Weight will be measured for all children <5 years of age using Seca 385 digital scales, with 20 g increment for weight below 20 kg and a 50 g increment for weight between 20 and 50 kg. Recumbent length will be measured for children <2 years of age using Seca 417 measuring boards with 1 mm increment. Standing height will be measured for children 2–5 years of age using Seca 213 portable stadiometers with 1 mm increment. Height and weight will be used to calculate height-for-age z-scores (HAZ) and weight-for-height z-scores (WHZ) based on WHO reference standards. A random subset of 10% of households will receive back check visits each day to repeat height/length measurements to ensure interobserver reliability.

#### Soil-transmitted helminth infection

Stool samples will be collected in rounds 2 and 4 from all household members in a randomly selected subset of 500 households and used to assess the presence and intensity of soil-transmitted helminth (STH) infection. Formalin ether concentration and microscopy will be used to quantify worms and ova for hookworms, *Ascaris lumbricoides*, and *Tricuris trichura.*^53^ Quality assurance includes independent duplicate assessment of all positive and 10% of negative samples. After stool collection, each participant will be offered a single dose of Albendazole, a broad-spectrum antihelmenthic drug recommended by the Ministry of Health and Family Welfare, Government of India. Stools collected in round 2 will allow for comparison of STH infection prevalence between intervention and control villages, while the stool samples collected ∼8 months later in round 4 will provide a measure of re-infection rate.

#### Environmental enteric dysfunction

Stools from a randomly selected subset of 200 children <2 years old, collected in rounds 2 and 4, will be used to assess EED through quantification of biomarkers of intestinal inflammation and permeability. Fecal myeloperoxidase (MPO), α-1-antitrypsin (AAT), and neopterin (NEO), markers for neutrophil activity, intestinal permeability and TH1 immune activation, respectively, were selected for this study based on evidence of association with EED, subsequent linear growth deficits and household environmental fecal contamination.^24^ ^25^ ^54^

#### Seroconversion for enteric pathogens

Serological assays that assess antibody production against various enteric pathogens can provide an objective measure of exposure to enteric infections.^55^ Enrolling children aged 6–18 months will reduce the potential for interference from maternally acquired antibodies and permit analysis of seroconversion data in a critical window for young children who experience higher diarrhoeal disease morbidity and mortality before 2 years of age.^56–61^ Children who are 6–12 months during round 2 will have capillary blood drawn by fingerstick or heelstick, as appropriate, and will be visited again during round 4 for a second capillary blood sample. All blood samples will be preserved on TropBio (Sydney, Australia) filter discs and stored within 7 days of collection at −20°C. Seroconversion against markers for norovirus, *Giardia intestinalis*, *Cryptosporidium parvum*, *Entamoeba histolytica*, enterotoxigenic *Escherichia coli*, heat-labile enterotoxin (ETEC-LT), *Salmonella* spp., *Campylobacter jejuni*, *Vibrio cholerae* and *Toxoplasma* spp. will be assessed using multiplex immunoassay technology on the Luminex xMAP platform.^62^

#### Environmental fecal contamination

Field workers will collect samples of household stored drinking water and source water from a random subset of 500 households in all four rounds, and child hand rinses in rounds 2 and 4. All water and hand rinse samples will be stored on ice during transport and analysed within 6 hours of collection using membrane filtration. Three assays will be used: (1) plating on m-Coli Blue 24 (Millipore, Billerica, Massachusetts, USA) for *E. coli* according to EPA Method 10 029, (2) alkaline peptone water enrichment prior to plating on thiosulfate citrate bile salts sucrose agar and slide agglutination serotyping for *V. cholerae* and (3) plating on xylose lysine desoxycholate agar, and slide agglutination serotyping for *Shigella* spp.^63–65^ Source and stored water samples will be assayed for *E. coli*, *V. cholerae* and *Shigella* spp., and hand rinse samples will be assayed for *E. coli* and *Shigella* spp. *E. coli* was selected as a standard non-human specific indicator of fecal contamination, though the limitations of this indicator are well-established.^66–68^ In order to better characterise human fecal contamination of the household environment, *V. cholerae* and *Shigella* spp. were selected based on prevalence in southern Asia, evidence of public health importance, and field laboratory limitations.^69–71^

#### Cost and cost-effectiveness

Costs and potential cost savings (ie, averted costs) associated with the intervention will be assessed through an economic costing approach that recognises and quantifies costs and benefits from a societal perspective.^72^ Data on programme and point-of-delivery inputs will be collected at household, community and implementer levels in round 3. Field workers will administer community surveys to a village leader, and household surveys to the household decision-maker for toilet installation, in 20 randomly selected households in 20 matched intervention and control villages. Given cost-effectiveness analyses require the effect of the intervention to be measured against a counterfactual, and the intervention of interest is a community-based intervention, cost and effectiveness measures will be summarised at the village level.^73^ Surveys will collect data on household-level and community-level inputs related to materials and labour required to construct household toilets and wash rooms, the community water tank and distribution system and household water connections; longer-term water supply and toilet maintenance costs and financing required for this infrastructure as well as perceived benefits, including averted social opportunity costs. Implementer inputs from Gram Vikas will be collected through an enumeration exercise, interviews and examination of the implementer's financial records.

#### Collective efficacy

CE is a latent construct comprised the structural and cognitive components that facilitate a community's shared belief in its ability to come together and execute actions related to a common goal.^21^ A review of the literature and established conceptual frameworks will be performed to define the CE construct. A sequential exploratory mixed qualitative and quantitative design will be used to develop and refine a scale to measure CE and test hypotheses. Field workers will administer the refined, multi-item, Likert-type CE scale to one randomly selected household member aged 18 years or older in each household in round 3.

#### Women's empowerment

Four dimensions of women's empowerment will be measured in rounds 3 and 4: group participation, leadership, decision-making and freedom of movement. Group participation and leadership will be measured using modules from the Women's Empowerment in Agriculture Index (WEAI), which has been tested in South Asia.^74^ Decision-making will be measured using questions from the women's status module of Demographic and Health Surveys. Freedom of movement will be measured using questions from the project-level Women's Empowerment in Agriculture Index (pro-WEAI). These measures will be collected for the primary female caregiver of the youngest child <5 years of age and were selected based on the importance of women's empowerment for child nutrition.^75^ ^76^ Women's empowerment is conceptualised as an outcome and a potential mediator along the pathway between the Gram Vikas intervention and child health outcomes.

#### Menstrual hygiene management

Menstrual hygiene management practices vary worldwide and depend on personal preference, socioeconomic status, local traditions and beliefs and access to water and sanitation resources.^77^ Unhygienic washing practices are common in rural India and among women and girls in lower socioeconomic groups and may increase risk of urogenital infection.^78–80^ However, the link between access to water and sanitation, menstrual hygiene management and urogenital infections has been poorly studied. Household surveys will be administered in round 4 to a randomly selected woman aged 18 or older, in a subset of 800 households, and will capture self-reported urogenital infection, defined as at least one of the following symptoms: (1) abnormal vaginal discharge (unusual texture and colour/more abundant than normal), (2) burning or itching in the genitalia, (3) burning or itching when urinating or (4) genital sores.^79^

#### Sanitation insecurity

This study will assess the associations between sanitation access and sanitation insecurity with mental health among women. In a previous research in Odisha, a contextually specific definition and measure for sanitation insecurity was developed, with associations between facets of sanitation insecurity and mental health independent of sanitation facility access.^81^ This previously developed measure will be used to determine if levels of sanitation insecurity differ between intervention and control villages and how it may be associated with mental health outcomes, specifically well-being, anxiety, depression and distress. Household surveys will be administered in round 4 to a randomly selected woman aged 18 or older, in a random subset of 800 households.

#### Fecal sludge management

In sanitation systems where sewerage is not feasible, such as the household toilets constructed as part of the MANTRA intervention, safe management of fecal waste is necessary. Although there is growing emphasis on safe fecal sludge management (FSM), research has mainly focused on urban settings.^82^ ^83^ Preliminary research in Odisha suggests that FSM in this rural setting is a substantial challenge and may impact household use of toilets. In round 3, household surveys and spot checks of toilets in intervention villages will be used to assess toilet use and FSM practices.

## Statistical analyses

The effect of the intervention on infrastructure coverage, access, and use (aim 1), and the effect of the intervention on improving health (aim 3), will be analysed using logistic, linear, log binomial or negative binomial multilevel regression depending on the outcome, to compare intervention versus control villages. Prevalence of FSM practices in intervention communities will be assessed using multilevel regression (aim 1). For all models, the hierarchical structure of the data will be accounted for using random effects. Estimation of relative risks through Poisson regression or binary regression methods for binary outcomes will be considered to ensure robustness of results. Mediation of the potential association between intervention and nutritional status outcomes by women's empowerment will be assessed using multilevel structural equation modelling, and statistical approaches to reduce bias will be explored as needed.^84^

The impact of intervention on reducing environmental fecal contamination (aim 2) will be assessed through two methods. First, hierarchical logistic and negative binomial multilevel regression to estimate intervention effects on the relative scale will be used to compare intervention versus control villages. Estimation of relative risks through Poisson regression or binary regression methods for binary outcomes will be considered to ensure robustness of results. Second, a stochastic microbial risk framework will be used to assess differential fecal environmental contamination between intervention and control villages.

The cost and cost-effectiveness of the intervention (aim 4) will be assessed in two steps. Incremental intervention benefits will be ascertained by combining health benefit data, from analysis of health outcome data and established averted cost data, with other averted social opportunity costs. An incremental cost-effectiveness ratio, expressed in cost per disease-specific DALY, will be calculated by dividing the incremental intervention costs by the incremental intervention benefits.

The CE scale will be analysed using a psychometric approach in which factor analytics are employed to identify an appropriate factor solution and test the reliability and validity of the CE scores. Once a CE factor solution and an empirically derived multilevel data structure have been identified, the association between CE and intervention effectiveness will be analysed using multilevel generalised linear mixed models to estimate relative risks,^85^ ^86^ (aim 5). For all outcomes, variables used in the matching process may be considered as covariates, as needed, in addition to individual, household and community-level risk factors. Covariates that are statistically associated with outcomes of interest in bivariate analyses will be considered for inclusion in final multivariable models, following standard stepwise model-building approaches. Secondary analyses may also evaluate models for effect modification as relevant, including exposure-mediator interaction for mediation models and cross-level interaction, by assessing changes in parameter values based on potential effect modifiers. Potential effect modifiers may include breastfeeding for seroconversion outcomes, and climate factors and population density for environmental fecal contamination and health outcomes. However, this study was not designed to assess effect modification and therefore is not specifically powered for these analyses. For all outcomes, unadjusted models will be presented along with models adjusting for covariates.

## Discussion

This matched cohort study is one of the first to evaluate the effect of a rural combined household-level piped water and sanitation intervention, implemented at the community level, on a large scale. The matched design provides a rigorous means for estimating causal effects given that randomisation to the intervention group was not feasible due to the several year implementation process.^5^ By focusing on an intervention where the implementation process is complete, it also limits the risk presented by randomised controlled trials, where the intervention has little uptake, an especially important study challenge given the interdependence of exposure and outcomes within communities, and a problem that has characterised previous trials of sanitation interventions in India.^15^ ^16^

A strength of this study is the assessment of health impacts using the holistic WHO definition of health, including not just disease status, and also mental, social and physical well-being.^87^ Outcomes along the causal chain include standard, but more subjective measures, such as reported diarrhoeal diseases and respiratory infection, as well as more objective measures such as fecal environmental contamination, soil-transmitted helminth infection and anthropometry. Although there is risk of response bias for reported outcomes, it is unlikely to be differential by intervention status since the study team is not directly linked to Gram Vikas. Even though field workers may be aware of village intervention status, laboratory staff analysing water, hand rinse, stool and blood samples will be blinded. In addition, this study includes the more novel use of seroconversion for enteric pathogens, biomarkers of EED and measures of CE in an evaluation assessment. While there are limitations inherent to observational studies, the matched study design and multivariable modelling analysis plan reduce the potential for confounding. However, there is still the potential for residual unmeasured confounding.

### Dissemination

Efforts will be made to communicate the central findings and implications with study communities, the implementing organisation and government officials in India. The results of this study will be submitted for publication in peer-reviewed journals and presented at conferences. The data collected in the study will be publicly available, with personal identifiable data redacted, following the publication of the primary results within 24 months of the final data collection date.
